# Case report: Axillary lymph node metastases from primary ovarian cancer: a report of two cases and literature review

**DOI:** 10.3389/fonc.2024.1384306

**Published:** 2024-05-21

**Authors:** Norlia Abdullah, Nadiah Rosly, Suria Hayati Md Pauzi, Aida Widure Mustapha, Yulianty Arifuddin

**Affiliations:** ^1^ Department of Surgery, Medical Faculty, Universiti Kebangsaan Malaysia, Kuala Lumpur, Malaysia; ^2^ Department of Surgery, Ipoh General Hospital, Ipoh, Perak, Malaysia; ^3^ Department of Pathology, Medical Faculty, Universiti Kebangsaan Malaysia, Kuala Lumpur, Malaysia; ^4^ Department of Radiology, Medical Faculty, Universiti Kebangsaan Malaysia, Kuala Lumpur, Malaysia; ^5^ Department of Obstetrics and Gynaecology, Medical Faculty, Universiti Kebangsaan Malaysia, Kuala Lumpur, Malaysia; ^6^ Department of Obstetrics and Gynaecology, An-Nur Specialist Hospital, Bangi, Selangor, Malaysia

**Keywords:** metastasis, axillary, lymph node, ovarian, carcinoma

## Abstract

Ovarian cancer is usually confined intraperitoneally. Distant metastases at presentation is unusual. Its spread via lymphatics is uncommon, and metastasis to axillary lymph nodes is very rare. We report two cases with presentation of axillary lymphadenopathy without breast involvement. Computed tomography scan identified the ovarian masses. Both had elevated Serum Ca 125. The first case had a Grade 2 ovarian endometrioid carcinoma. The second case had a high-grade serous ovarian carcinoma. These cases illustrate the rarity of axillary lymphadenopathy from ovarian cancer. It is important to identify the primary ovarian carcinoma in order to offer appropriate management. Despite surgery and chemotherapy, both succumbed within 3 years from diagnosis.

## Introduction

In 2022, the incidence of ovarian cancer was reported to be 324,602 cases worldwide resulting in 206,956 deaths (1). Ovarian cancer has been identified to be the most common gynecological cancer (2). It is usually diagnosed at the late stage due to its lack of clinical presentation until peritoneal advancement is apparent (3), and most patients die within 5 years from initial diagnosis (4). Ovarian cancer causes the highest number of deaths compared to other gynecological cancers (5). Distant metastasis beyond the abdominal cavity is rare at the initial presentation. The most common lymph node metastases from ovarian carcinoma are to abdominal, para-aortic, mediastinal, and pelvic structures. From our literature review, axillary lymph node metastases are very rare and only a few cases have been reported (6). We report two cases of women with ovarian cancer who presented with axillary lymph node metastases.

The aim of this report is to bring awareness to the rare but possible occurrence of axillary lymph node metastasis arising from ovarian carcinoma. Thus, computed tomography (CT) staging for such cases should include the pelvis and not just stop at the abdominal region. Treatment will then need to be directed to the source of the malignancy.

## Case reports

### Case 1

A 54-year-old Para 3, with no family history of gynecological malignancy presented with a 1-year history of abdominal distension and a self-noted mass in the lower abdomen. She was an obese woman (BMI: 30.5 kg/m^2^), menarche at 14 years old, first childbirth at 28 years old, and menopause at 52 years old. She did not consume any contraceptive pills or hormone replacement therapy and did not smoke or consume alcohol. She had no previous history of endometriosis. There was no family history of breast and/or ovarian carcinoma.

At an earlier clinic, she was diagnosed to have gastritis and given the relevant medication. Only at a second medical center when she presented several weeks later was a thorough examination done that revealed a large abdominal mass with a small right axillary palpable mass. Her serum tumor marker Ca 125 was raised to 309 U/ml (normal value <35 U/ml). Ultrasonography detected a large pelvic mass likely to be ovarian in origin. Computed tomograph scans of the thorax, abdomen and pelvis demonstrated a right ovarian mass with minimal ascites and the presence of pelvic and right axillary lymphadenopathy. The ascitic fluid did not contain any malignant cells. Mammograms did not reveal any suspicious breast lesions. Ultrasound of the axillas however showed a lobulated right axillary lymph node with loss of normal fatty hilum measuring 2.5 cm × 1.5 cm. Ultrasound-guided biopsy of the right axillary lymph node was performed. The histology of the right axillary lymph node was metastatic adenocarcinoma with possible primary sites arising from the breast, ovary, or endometrium. The patient was referred to the gynecology oncology team for evaluation of the ovarian mass. She underwent a staging laparotomy consisting of a total abdominal hysterectomy, bilateral salpingo-oophorectomy (TAHBSO) and left pelvic lymphadenectomy for suspected ovarian cancer stage 1C. Intraoperative findings were a left ovarian mass measuring 25 cm × 25 cm, with minimal ascites and adhesion of the posterior part of the tumor to the bowel. The right ovary was atrophic. The omentum and appendix were normal.

Histology of the right axillary lymph node revealed tumor cells exhibiting moderately pleomorphic vesicular nuclei with many prominent nucleoli and occasional mitosis. Immunohistochemistry testing was positive for cytokeratin 7 (CK7) and estrogen receptor (ER), but negative for CK20. This was concluded as metastatic adenocarcinoma with possible primary sites of breast, ovarian, and endometrium. Histology of the left ovary was reported as an endometrioid carcinoma (FIGO grade II, pT1a, N0Mx). No evidence of malignancy in the other organs and left pelvic lymph nodes. Immunohistochemistry result was malignant cells positive for ER, progesterone receptor (PR), p16, and p53, and negative for Wilms Tumor Gene 1 (WT-1). She was advised to undergo chemotherapy and radiotherapy but declined and defaulted follow-up.

She only returned 2 years later with a mass in the right axilla that was associated with pain and discharge. There was a large fungating lesion extending from the right axilla to the upper outer quadrant of the breast with subcutaneous tissue thickening. A repeat histology of the axillary mass revealed a metastatic adenocarcinoma with the primary most likely to be the ovary. She was offered palliative chemotherapy, but declined again. The ulcerated axillary mass required repeated wound debridement and regular dressing. She finally agreed to undergo chemotherapy only a year later. However, the lesion did not respond after four cycles of chemotherapy (Carboplatin 620 mg, Paclitaxel 280 mg) despite a reducing trend of serum CA125. Subsequently, she received 13 fractions of radiotherapy, but again the lesion remains unchanged. As the tumor was positive for estrogen receptor, she was started on letrozole. Surprisingly, within 4 months, the axillary lesion responded by reduction of 10%–15% in size. Unfortunately, she expired 4 months later due to pneumonia, 3 years from the initial presentation.

### Case 2

A 51-year-old nullipara presented with a left axillary swelling with intermittent discomfort for a month. She attained menarche at 12 years of age and was perimenopausal with no previous history of endometriosis. Her body mass index was 28.1 kg/m^2.^ She did not smoke or drink alcohol. She did not consume any contraceptive pills or hormone replacement therapy and did not smoke or consume alcohol. She had no previous history of endometriosis. There was no family history of breast and/or ovarian carcinoma.

She had severe headache and vertigo 3 months prior and was diagnosed to have hypertension at another clinic. The medication brought some relief. Examination revealed a 5 cm × 3 cm hard but mobile left axillary lymph node. As there was no evidence of malignancy from the mammogram and ultrasound of the breast, the provisional diagnosis then was an occult left breast carcinoma. She underwent left axillary dissection. Histological examination revealed a high-grade serous carcinoma of ovarian or uterine origin. Immunohistochemical studies reported those malignant cells were positive for CK 7, ER, CA 125, and p53 with focal positivity toward WT-1, negative for CK 20 and vimentin.

CT scan of the thorax, abdomen, and pelvis showed a right ovarian mass with lymphadenopathy at the para-aortic, mesenteric, left axilla, and uterine cervix. There was no ascites. Serum tumor marker Ca 125 was raised at 502 U/ml. The intra-operative findings were a right ovarian tumor measuring 10 cm × 10 cm, cystic with solid components adhering to the small bowel, with multiple enlarged peritoneal nodules and para-aortic lymph nodes. TAHBSO, debulking and segmental small bowel resection with primary anastomosis were done. The histological report was a high-grade serous right ovarian carcinoma with focal capsular breach, with malignant cells seen in the right fallopian tube wall, right posterior uterine wall with metastases to the omentum, distal ileum and mesentery. (TNM stage: T2a, Nx, Mx, and FIGO II). Immunohistochemical studies showed these malignant cells were the same as that of the left axillary lymph node. She received five cycles of carboplatin 450 mg, paclitaxel 270 mg, and bevacizumab 450 mg. However, the patient did not proceed with further treatment due to financial constraints. Her vertigo worsened with nystagmus but the MRI brain was negative for metastases. She expired shortly afterwards, 2.5 years from her initial presentation.

**Figure 1:**
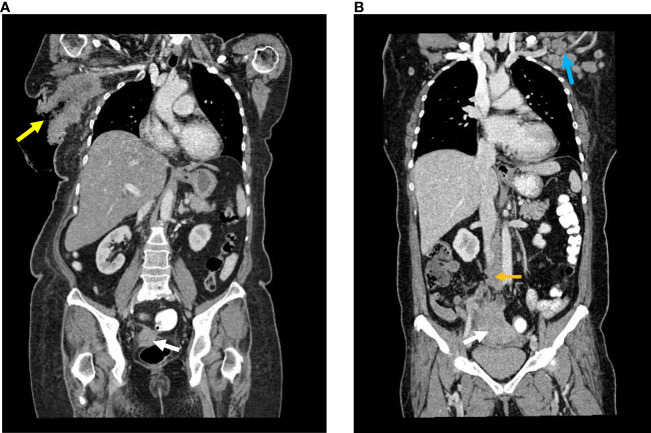
**(A)** Case A Reformatted coronal image of contrasted CT : Enhancing lesion arising at the right adnexa with no clear fat plane with the rectum (white arrow). Large right axillary mass with ulceration (yellow arrow). **(B)** Case B Reformatted coronal image of contrasted CT : Large mass arising from the right adnexa (white arrow), enlarged retroperitoneal lymph nodes (yellow arrow) and enlarged left axillary lymph node (blue arrow).

The coronal CT images are shown in **Figures 1A, B**.

**Figure 2:**
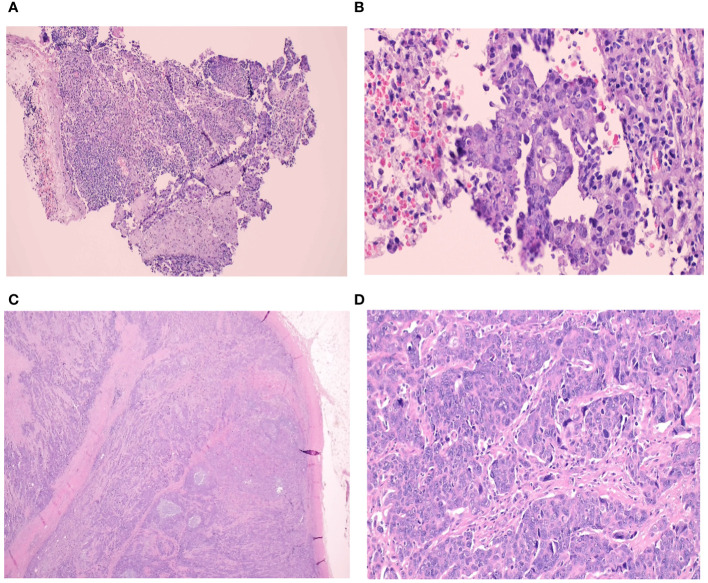
**(A-D)** Histological slides. Core needle biopsy of the right axillary lymph node from Case. A **(A)** Foci of metastatic carcinoma are observed in a lymphoid background (H&E, 10x) **(B)** This metastatic tumour forms glands lined by malignant cells with pleomorphic nuclei and prominent nucleoli. These morphological features are similar to the main endometrioid carcinoma in her left ovary (H&E, 40x) A left axillary lymph node core biopsy from the second case. **(C)** The lymph node is fully-effaced with infiltration by metastatic carcinoma (H&E, 1.25x) **(D)** The malignant cells are arranged in clusters and display pleomorphic nuclei with conspicuous nucleoli. The morphology and immunoprofile is consistent with serous carcinoma. Similar tumour is seen in her right ovary (H&E, 40x).

The pathology slides are shown in **Figures 2A–D**.

## Discussion

Based on the National Cancer Registry in 2012–2016, ovarian cancer is the 10th commonest cancer in Malaysia but the 5th commonest cancer among women. The most common cancers in Malaysian women are breast followed by colorectal, cervix, and lung. Ovarian cancer makes up 3.1% of all reported cancers with an age standardized rate of 5.6 per 100,000 population. In comparison to earlier data in 2007–2011, ovarian cancer then was the 9th commonest cancer in Malaysia contributing to 3.4% of all reported cancers with an age-standardized rate of 5.9 per 100,000 population (7).

Ovarian cancer is a major cause of mortality in women as it is a deadly disease with a curative rate of only 30% (8). In a study done in Universiti Sains Malaysia, the median survival time for women with ovarian cancer was 38 months with a 5-year survival probability of 35.2% (9). There is currently no standard ovarian screening tool (10). Recent data from two large UK trials have shown that multimodal ovarian cancer screening using a longitudinal CA 125 algorithm has resulted in diagnosis at an earlier stage, both in average and high-risk women. However, there has been no randomized controlled trial that demonstrated a definitive mortality benefit. Screening is not currently recommended in the general population (11).

The actual cause of ovarian carcinoma has not been identified. The identified risk factors are genetic abnormalities (BRCA 1 and BRCA 2), a positive family history of breast and ovarian carcinoma, nulliparity, increasing age, and tall women. Researchers hypothesize that the same hormones that make women grow taller may at the same time increase the chance of dividing cells becoming abnormal and turn malignant (12).

Ovarian cancer has subtypes consisting of epithelial, sarcoma, germ cell, and stromal tumor with epithelial type being the most common. Distant metastasis is unusual at initial presentation. Its most common mean of spread is intraperitoneally, while local invasion, lymphatic invasion and hematogenous spread are less common. Its most common sites of metastases to visceral organs are liver, lung, and pleura, and less commonly to the central nervous system, bone, skin, spleen, and breast (6). Whereas the common site for lymph node spread is peritoneally to abdominal (47%), para-aortic (38%), mediastinal (29%), and pelvic (17%) lymph nodes. Cormio et al. (6) reported that only 8% of their patients were initially diagnosed with distant metastases, whereas 22% developed distant metastases during their course of illness. Regardless of the histologic type of ovarian cancer, its metastatic pattern is uniform with peritoneal spread being the most common site of metastases (13).

Primary ovarian cancer with axillary lymph node metastases is a rare occasion. Axillary lymphadenopathy is most often associated with breast carcinoma which is seen in 40% of patients at initial presentation. Axillary metastases from ovarian cancer often also present with breast metastases and only occasionally seen without involvement of the breast. Serous ovarian cancer is the most common type of ovarian cancer to metastasize to the breast (14).

One pathogenesis of the spread of the ovarian carcinoma to axillary lymph nodes is when the malignant cells are present in ascitic fluid and peritoneal carcinomatosis has occurred. This will lead to transdiaphragmatic invasion to the superior diaphragmatic lymph nodes. Then, anterior to the prepericardial lymph nodes to the internal jugular and subclavian veins or to the subclavian lymph trunk into axillary lymph nodes. Another route is invasion into lymphatic vessels which form the cisterna chyli and thoracic duct into the axillary lymph nodes (15). The thoracic duct begins in the abdomen, just inferior to the diaphragm, as an elongated lymph sac, which is called the cisterna chyli. The cisterna chyli lies to the right of the aorta and receives the intestinal trunk, the right and left lumbar trunks and lymphatic vessels at the lower part of the thorax. The internal iliac nodes, external iliac nodes, common iliac nodes and para-aortic nodes join the lumbar trunks on the posterior abdominal wall (16). It would be reasonable to postulate that as ovarian lymphatic drainage flows toward the para-aortic and paracaval lymph nodes as described by Kleppe et al (17), malignant cells would be able to metastasis from the ovaries to the supradiaphragmatic lymph nodes, including the axillary lymph nodes. This could occur even in the absence of significant ascites.

When axillary lymphadenopathy is seen without breast involvement, the first clinical diagnosis would be an occult breast carcinoma (18). The basis of diagnosis largely depends on the intraoperative findings, histological and immunohistochemistry reports, and tumor marker levels. Immunohistochemistry plays a major part in distinguishing the primary site of disease. Gross Cystic Disease Fluid Protein 15 is a protein available in breast tissue and is a marker for apocrine cells and breast cancer cells with apocrine differentiation, which is highly sensitive in detecting breast cancer. Its sensitivity is reported to be 74% and specificity of 95% (19). Whereas another marker used is WT-1, which is a tumor suppressor gene which is seen during development of reproductive organs. A research by Al-Hussaini et al. (20) significantly showed WT-1 to be positive in 94.7% of ovarian serous carcinoma and 100% of peritoneal serous carcinoma.

The standard treatment for ovarian carcinoma consists of cytoreductive surgery, platinum-based chemotherapy in combination with bevacizumab, followed by bevacizumab only for 18 months (15). For many, especially in developing and underdeveloped countries, access to bevacizumab is often not possible due to its high cost, thus compromising the patients’ outcome.

Our first patient with endometrioid ovarian carcinoma has a less aggressive carcinoma compared to serous carcinoma (21). In addition to it being ER positive, this may explain why it responded fairly well despite given oral letrozole for only 4 months (22).

In a systematic review paper by Koufopoulos et al. (23), they identified 25 cases in a total of 21 manuscripts, of ovarian carcinoma with axillary lymph node metastasis spanning from 1997 to 2023. With the presence of these metastsasis, the cases were classified as Figo Stage 4b. The presentations were divided into two types:

1) Axillary lymphadenopathy at the onset (eight cases)
2) Axillary lymphadenopathy several years later while on follow-up after an earlier diagnosis of ovarian cancer (17 cases).

Ascites was stated to be present in only six of the 25 cases. The presence of malignant cells was found in the ascitic fluid in three cases. The ovarian carcinoma was serous in 24 of the 25 cases. Four cases underwent neoadjuvant chemotherapy followed by debulking surgery. The rest had surgery followed by adjuvant chemotherapy. There was no difference in survival comparing the women of the above two types of presentation. The median disease-free survival was reported to be 12 months.

In Malaysia, a developing country, as in other places, detection of ovarian carcinoma in the early stages is challenging. To make matters worse, a significant number of patients may decline or delay standard treatment once a diagnosis is made, in favor of traditional or alternative treatment as was the situation in Case 1 (24).

## Conclusion

These cases depict rare occurrences of axillary metastases from ovarian origin. Multiple modalities are used to detect the disease and its metastases, more importantly the histology and immunohistochemistry testing. Despite the rarity of these patterns of disease, a high index of suspicion is needed in order to make the correct diagnosis, which is necessary to deliver the appropriate treatment.

## Data availability statement

The original contributions presented in the study are included in the article/supplementary material. Further inquiries can be directed to the corresponding author.

## Ethics statement

Written informed consent was obtained from the individual(s) for the publication of any potentially identifiable images or data included in this article. Written informed consent was obtained from the participant/patient(s) for the publication of this case report.

## Author contributions

NA: Conceptualization, Funding acquisition, Supervision, Writing – review & editing. NR: Writing – original draft. SMP: Investigation, Writing – review & editing. AM: Investigation, Writing – review & editing. YA: Writing – review & editing.
